# Radiomics based on readout-segmented echo-planar imaging (RS-EPI) diffusion-weighted imaging (DWI) for prognostic risk stratification of patients with rectal cancer: a two-centre, machine learning study using the framework of predictive, preventive, and personalized medicine

**DOI:** 10.1007/s13167-022-00303-3

**Published:** 2022-11-12

**Authors:** Zonglin Liu, Yueming Wang, Fu Shen, Zhiyuan Zhang, Jing Gong, Caixia Fu, Changqing Shen, Rong Li, Guodong Jing, Sanjun Cai, Zhen Zhang, Yiqun Sun, Tong Tong

**Affiliations:** 1 Department of Radiology, Fudan University Shanghai Cancer Center, Shanghai, China; 2 Department of Oncology, Shanghai Medical College, Fudan University, Shanghai, China; 3 Department of Anatomy and Physiology, Shanghai JiaoTong University School of Medicine, Shanghai, China; 4 Department of Radiology, Changhai Hospital, Second Military Medical University, Shanghai, China; 5 Department of Radiation Oncology, Fudan University Shanghai Cancer Center, Shanghai, China; 6 MR Application Development, Siemens Shenzhen Magnetic Resonance Ltd, Shenzhen, China; 7 Department of Colorectal Surgery, Fudan University Shanghai Cancer Center, Shanghai, China

**Keywords:** Readout-segmented echo-planar imaging, Diffusion-weighted imaging, Rectal cancer, Recurrence or metastasis, Machine learning, Predictive preventive personalized medicine

## Abstract

**Background:**

Currently, the rate of recurrence or metastasis (ROM) remains high in rectal cancer (RC) patients treated with the standard regimen. The potential of diffusion-weighted imaging (DWI) in predicting ROM risk has been reported, but the efficacy is insufficient.

**Aims:**

This study investigated the potential of a new sequence called readout-segmented echo-planar imaging (RS-EPI) DWI in predicting the ROM risk of patients with RC using machine learning methods to achieve the principle of predictive, preventive, and personalized medicine (PPPM) application in RC treatment.

**Methods:**

A total of 195 RC patients from two centres who directly received total mesorectal excision were retrospectively enrolled in our study. Machine learning methods, including recursive feature elimination (RFE), the synthetic minority oversampling technique (SMOTE), and the support vector machine (SVM) classifier, were used to construct models based on clinical-pathological factors (clinical model), radiomic features from RS-EPI DWI (radiomics model), and their combination (merged model). The Harrell concordance index (C-index) and the area under the time-dependent receiver operating characteristic curve (AUC) were calculated to evaluate the predictive performance at 1 year, 3 years, and 5 years. Kaplan‒Meier analysis was performed to evaluate the ability to stratify patients according to the risk of ROM.

**Findings:**

The merged model performed well in predicting tumour ROM in patients with RC at 1 year, 3 years, and 5 years in both cohorts (AUC = 0.887/0.813/0.794; 0.819/0.795/0.783) and was significantly superior to the clinical model (AUC = 0.87 [95% CI: 0.80–0.93] vs. 0.71 [95% CI: 0.59–0.81], *p* = 0.009; C-index = 0.83 [95% CI: 0.76–0.90] vs. 0.68 [95% CI: 0.56–0.79], *p* = 0.002). It also had a significant ability to differentiate patients with a high and low risk of ROM (HR = 12.189 [95% CI: 4.976–29.853], *p* < 0.001; HR = 6.427 [95% CI: 2.265–13.036], *p* = 0.002).

**Conclusion:**

Our developed merged model based on RS-EPI DWI accurately predicted and effectively stratified patients with RC according to the ROM risk at an early stage with an individualized profile, which may be able to assist physicians in individualizing the treatment protocols and promote a meaningful paradigm shift in RC treatment from traditional reactive medicine to PPPM.

**Supplementary Information:**

The online version contains supplementary material available at 10.1007/s13167-022-00303-3.

## Introduction

### Correlation between predictive, preventive, and personalized medicine (PPPM) and current research

As the perspective of predictive, preventive, and personalized medicine (PPPM) has developed and become prevalent, emphasis on the treatment of cancer based on more precise stratification of patients according to the multiple biological characteristics of the tumour has increased, leading to further improvements in personalized treatment plans and achieving a better prognosis for patients [1]. Rectal cancer (RC) is a globally prevalent malignancy, and its high recurrence rate is a major cause of its significant mortality [2]. Although the risk of local recurrence or distant metastasis (ROM) after curative surgery has been significantly reduced with the continuous optimization of standard guidelines for adjuvant therapies such as radiation and chemotherapy, it still occurs in approximately 30% of RC patients [3, 4]. More importantly, the interval between initial treatment and recurrence is an important prognostic factor, and inappropriate integration of adjuvant therapy may result in a worse prognosis [5], which requires an accurate prediction of the ROM risk of patients at an early stage. Within the framework of the current standard guidelines, the lag in the acquisition of proven pathological risk factors has resulted in the treatment paradigm for RC still leaning towards reactive medicine. Therefore, the discovery of a more effective biomarker for predicting long-term prognosis prior to treatment is urgently needed to provide a valid reference for the early adjustment of treatment regimens and prevention of inappropriate therapies, consistent with the goal of PPPM.

### Radiomics with machine learning (ML) is an effective PPPM-based tool for the prediction and targeted treatment of RC

Benefiting from the noninvasive feature, magnetic resonance imaging (MRI) was considered a routine examination to assess the tumour location, morphological characteristics, and lymph node metastasis for the evaluation of TNM staging and was used to provide prognostic information [6–8]. As mentioned in the white paper of the “European Association for predictive, preventive, and personalized medicine” (PPPM), MRI was a key predictive companion biomarker that might improve the benefit of treatment for previously diagnosed diseases through a risk stratification of patients. Currently, the methodology of radiomics combined with ML algorithms is widely used in the construction of predictive models that have been used to mine and incorporate additional information from the large amounts of objective quantitative features extracted that are not perceived using conventional methods. The potential of ML in feature selection, prognostic biomarker development, targeted management, and personalized medicine has been reported [9–16].

### Improvements in image quality may facilitate the development of more PPPM-compliant biomarkers

As one of the most frequently utilized sequences in MRI scanning, diffusion-weighted imaging (DWI) has been reported to be valuable in determining the prognosis [17]. DWI radiomics features were reported to effectively predict the treatment outcome and long-term prognosis of patients with RC, including downstaging of clinical T and N stages, pathological complete response (PCR), overall survival (OS), and disease-free survival (DFS).

Currently, single shot echo-planar imaging (SS-EPI) is the most commonly used technique for DWI. Readout-segmented echo-planar imaging (RS-EPI) DWI is a new technique that splits the K-space into multiple segments to obtain a shorter echo space along the readout direction [18] and has been reported to significantly increase the resolution and quality of images of various tumours, including RC [19]. However, the utilization of RS-EPI DWI in constructing radiomics prediction models has rarely been reported. To the best of our knowledge, its role in assessing the risk of tumour ROM remains unclear. We hypothesize that improvements in image quality may enable biomarkers to be more representative of the true characteristics of tumours, contribute to improving the predictive performance of the biomarkers, and fuel the development of PPPM in RC therapy.

Therefore, our study proposed the extraction of radiomic features based on ADC maps derived from RS-EPI in the framework of PPPM, establishment of an optimal radiomics model with ML to explore the potential for predicting the prognosis, and further analysis of the incremental value of the model combining the clinicopathologic and radiomic features compared to the model using the clinicopathologic factors alone.

## Methods

### Patients

**Fig. 1:**
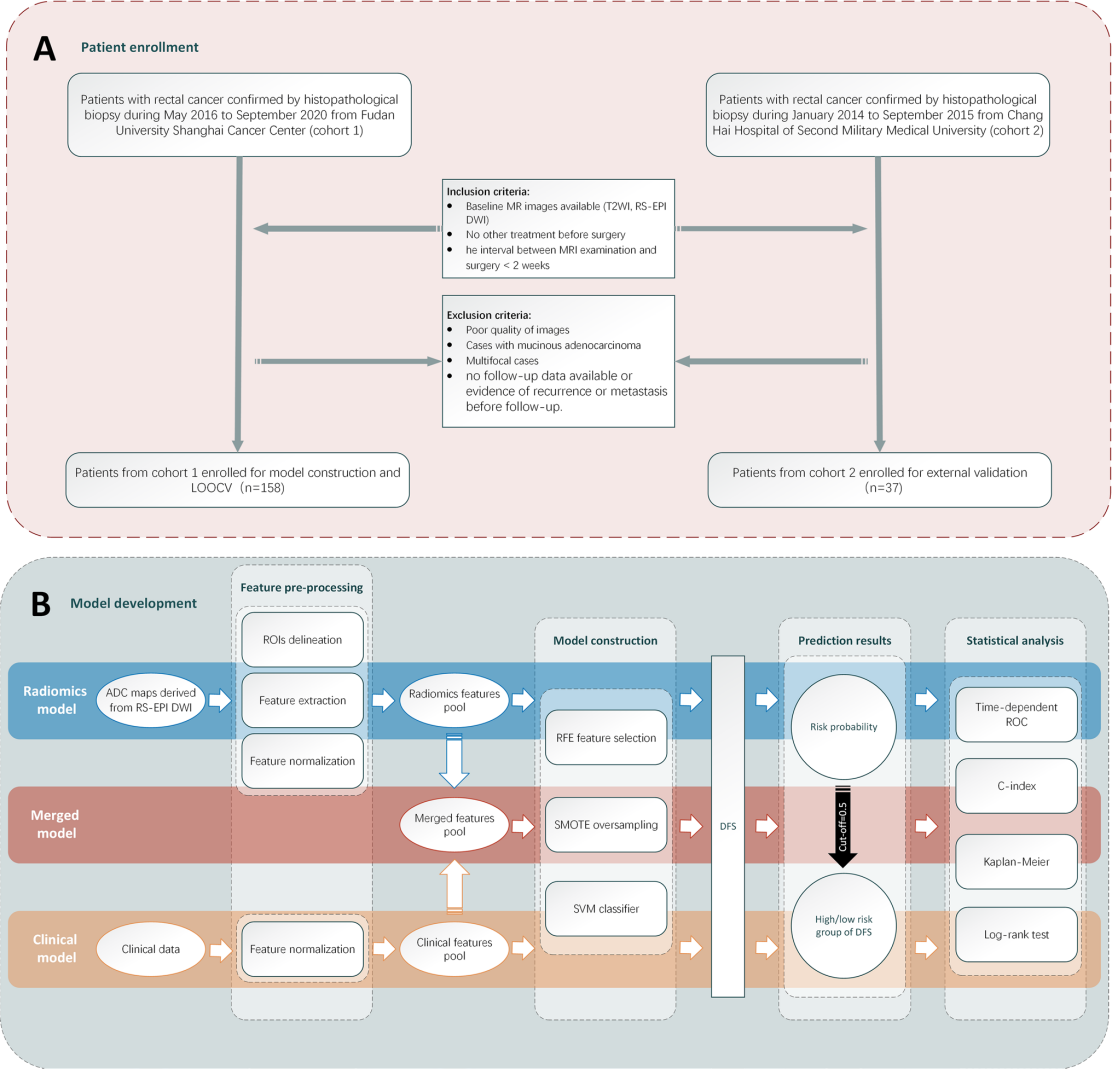
**a** Flow chart of the patient selection and enrolment process. **b** General process used to construct our proposed model for the prediction of DFS in patients with RC. Abbreviations: LOOCV, leave-one-out cross-validation; RS-EPI DWI, readout-segmented echo-planar imaging; DWI, diffusion-weighted imaging; ADC, apparent diffusion coefficient; ROIs, regions of interest; RFE, recursive feature elimination; SMOTE, synthetic minority oversampling technique; SVM, support vector machine; DFS, disease-free survival; ROC, receiver operating characteristic

In this retrospective study, patients with RC confirmed by histopathological biopsy from January 2014 to September 2020 were consecutively enrolled from two centres. The inclusion criteria were as follows: (1) baseline MR images available, including T2-weighted images (T2WI), RS-EPI DWI, and derived ADC maps; (2) no other treatment was received in the period between the baseline MR scans and surgical resection; and (3) the interval between the MRI examination and surgery was not more than 2 weeks. We performed postoperative follow-up for all patients who met the aforementioned criteria, with the endpoint of reaching the deadline or the occurrence of ROM. The exclusion criteria were as follows: (1) excessively poor-quality images leading to an inability to differentiate the lesions accurately; (2) patients with mucinous adenocarcinoma; (3) patients with multifocal disease; and (4) no follow-up data were available or evidence of ROM was observed before follow-up. The detailed process of recruitment is presented in Fig. 1a.

In addition, baseline clinicopathologic characteristics, including carcinoembryonic antigen (CEA), carbohydrate antigen 199 (CA-199), pathological T stage (pT stage), pathological N stage (pN stage), tumour differentiation, intravenous tumour emboli, perineural invasion (PNI), and whether the patient underwent postoperative adjuvant therapy (radiation or chemotherapy), were collected from the electronic medical records system.

The patients were arranged into two cohorts: (1) the patients from Fudan University Shanghai Cancer Center were included as the training and internal validation cohorts (cohort 1); and (2) the patients from Chang Hai Hospital of Second Military Medical University were included as the external validation cohort (cohort 2).

### MRI protocols and image segmentation

None of the patients were subjected to any bowel preparation prior to the MRI examination. The protocols mainly comprised sagittal and oblique axial T2WI with the turbo spin‒echo technique and RS-EPI DWI in oblique axial orientation. The detailed parameters of the MRI scanners for the two centres are presented in Table S1 in the Supplement. After completing scanning, all series of required images were exported from the Picture Archiving and Communication System to the personal workstation in Dicom format. Next, *ITK-SNAP* (version 3.8.0, www.itk-snap.org) software was used to delineate the regions of interest (ROIs) in each image. Two radiologists (LZL and SYQ, with 1 and 9 years of radiodiagnosis experience in RC) manually outlined the lesion layer by layer on oblique axial ADC maps derived from RS-EPI DWI with reference to T2WI, during which they were blinded to the clinicopathological information and had no communication. When encountering cases of uncertainty or difficulty in outlining, the decision was determined by discussion to achieve a consensus, endeavouring to cover the whole lesions and crop the regions of cysts and necrosis. Eventually, a corresponding 3-dimensional mask was generated for each lesion.

### Image analysis and model development

In our study, cohort 1 was used to construct three ML-based models for predicting the DFS of patients with RC separately, and the models applied the radiomic features, clinicopathological factors, and the convergence of the two features (merged model). We used the same method to construct the three models as an approach to minimize the effects of differences in the modelling method on the predictive performance and to increase the comparability between each model. The predicted outcome is represented by the model-calculated risk probability (*prob*) of ROM. In addition, the performance of the merged model was further validated in cohort 2. The general process of model construction is shown in Fig. 1b, and details are described in subsequent sections.

Then, the open-source *Python* package *PyRadiomics* (http://pyradiomics.readthedocs.io/, v1.3.1) was used to extract the radiomic features from the RS-EPI DWI-derived ADC maps, and ML algorithms were applied to build the model for the classification of the DFS status. Feature extraction was implemented based on three types of images: *Original*; *LoG* with sigma values of 3, 4, and 5; and *Wavelet.* Each type of image consisted of six categories of features: shape-based, first-order statistics, grey level cooccurrence matrix [*GLCM*], grey level run length matrix [*GLRLM*], grey level size zone matrix [*GLSZM*], and grey level dependence matrix [*GLDM*]. The value of the resampled pixel spacing was set to [1.5, 1.5, 5], the image discretization bin width was set to 5, and the voxel array shift was 300.

After completing radiomic feature extraction, the open-source *Python* package *scikit-learn* (https://scikit-learn.org/stable/, v1.1.1) was used for data processing and model development. The Z score of each image feature extracted from the ROI was calculated separately using the preprocessing method in the *StandardScaler* function to ensure the comparability of the dynamic range of radiomic features before selection. Support vector machine (SVM) regression and recursive feature elimination (RFE) feature selection methods were used to select the optimal features and address the problem of a large number of redundant features in the initial feature library. Furthermore, the synthetic minority oversampling technique (SMOTE) was used to oversample the minority data to improve the balance of our dataset, which was required for the robustness of the model. For the establishment of the classification model, the SVM classifier was utilized. Furthermore, the leave-one-out cross-validation (LOOCV) method was applied to train and validate the classifier performance in every patient in cohort 1. The SVM classifier and oversampling process were embedded into the cycles of LOOCV training/validation, and artificial tumours calculated by the SMOTE algorithm were integrated only in the process of training to prevent bias in data segmentation.

Baseline CEA levels, CA-199 levels, pT stage, pN stage, tumour differentiation, intravenous tumour emboli, PNI, and whether the patient underwent postoperative adjuvant therapy were included after quantification and normalization to dichotomous variables with the predetermined standard to build a clinical model for predicting the ROM risk of patients with RC. The *StandardScaler*, SVM regression, RFE feature selection, SMOTE, SVM classifier, and LOOCV methods were also applied in the development of the clinical model.

We merged all radiomic features and clinicopathological factors to build a merged model as a method to investigate whether combining radiological information with clinicopathological characteristics improved the predictive performance. We first included all of the initial clinicopathological factors and original radiomic features into one feature pool and then implemented SVM regression and RFE feature screening. The other methodology used for model construction is consistent with the methods described above.

Because the LOOCV method feature of every cycle of LOOCV training/validation distribution was not the same, we included all patients from cohort 1 into the constructed merged model as the training cohort and then validated the performance of the fitted model using the patients from cohort 2.

### Statistical analysis

All statistical analyses were performed with *SPSS* (version 26.0; Chicago, Ill) and *R* (version 4.2.0; http://www.Rproject.org). *p* values less than 0.05 were recognized as statistically significant. Numerical values were counted and are presented as the median values and interquartile ranges (IQRs), while categorical variables are presented as frequencies and percentages. Patients were divided into high-risk and low-risk groups based on a cut-off value for the calculated risk probability to evaluate the prediction performance of each model, including the RS-EPI DWI-derived ADC map-based model, clinical model, and merged model, and 0.5 was considered the optimal threshold. The time-dependent receiver operating characteristic (time-ROC) with the area under the ROC curve (AUC) value were used to evaluate the prediction performance of each model at the time points of 1, 3, and 5 years, and the DeLong test was used to evaluate the difference in the overall AUC value between the models. The Harrell concordance index (C-index) of each model for each year was calculated and visualized by constructing a curve, and the *p* value of the overall C-index comparison between the models was calculated to reflect the difference in predictive performance. Decision curve analysis (DCA) was used to evaluate clinical utility. A calibration curve was constructed to evaluate the accuracy of the predictive results. Kaplan‒Meier (K-M) survival curves with risk tables and log-rank tests were used to compare the status of ROM between the high-risk group and the low-risk group, and hazard ratios (HRs) were simultaneously calculated. The corresponding 95% confidence interval (CI) was simultaneously calculated using each statistical method described above.

## Results

### Patient characteristics

A total of 195 patients meeting these criteria were selected for further analysis: 158 in cohort 1 and 37 in cohort 2. The detailed baseline clinicopathological characteristics of the patients included in the study from the two cohorts are summarized in Table 1. Except for age and intravenous tumour emboli, no significant differences were observed between the two cohorts.

**Table 1:** Comparison of baseline clinicopathologic characteristics between patients of two centres. Patient baseline characteristics

Characteristic	Fudan University Shanghai Cancer Centre, *N* = 158^1^	Chang Hai Hospital of Second Military Medical University, *N* = 37^1^	*p* value^2^
Age	66.0 (57.0, 72.0)	58.0 (49.0, 65.0)	< 0.001
Sex			0.714
Female	70 (44.3%)	18 (48.6%)	
Male	88 (55.7%)	19 (51.4%)	
CEA			0.419
≤ 5 ng/ml	117 (74.1%)	25 (67.6%)	
> 5 ng/ml	41 (25.9%)	12 (32.4%)	
CA19-9			0.244
≤ 37u/ml	143 (90.5%)	31 (83.8%)	
> 37u/ml	15 (9.5%)	6 (16.2%)	
Tumour differentiation			0.485
Median-high	126 (79.7%)	32 (86.5%)	
Low	32 (9.5%)	5 (14.5%)	
pT stage			0.193
pT1-2	67 (42.4%)	11 (29.7%)	
pT3-4	91 (57.6%)	26 (70.3%)	
pN stage			0.194
pN0	100 (63.3%)	19 (51.4%)	
pN1-2	58 (36.7%)	18 (48.6%)	
Intravenous tumour emboli			0.001
( −)	104 (65.8%)	34 (91.9%)	
( +)	54 (34.2%)	3 (8.1%)	
PNI			0.117
( −)	121 (76.6%)	33 (89.2%)	
( +)	37 (23.4%)	4 (10.8%)	
Post-operational adjuvant therapy			> 0.999
( −)	79 (50.0%)	18 (48.6%)	
( +)	79 (50.0%)	19 (51.4%)	
Recurrence or metastasis			0.505
( −)	127 (80.4%)	28 (75.7%)	
( +)	31 (19.6%)	9 (24.3%)	
DFS	29.0 (17.0, 48.0)	83.0 (24.0, 95.0)	< 0.001

^1^Median (IQR); *n* (%)

^2^Welch two-sample *t*-test; Fisher’s exact test

Abbreviations: a. CEA: carcinoembryonic antigen, b. CA19-9: carbohydrate antigen 19–9, c. pT stage: pathological T stage, d. pN stage: pathological N stage e. PNI: perineural invasion, f. DFS: disease-free survival

### Model construction and information

Three models were constructed, including a clinical model, a radiomics model, and a merged model, for predicting the status of DFS. We extracted 1046 features from the RS-EPI DWI-derived ADC maps for each patient, including 100 *Original* features, 258 *LoG* features, and 688 *Wavelet* features. Finally, 15 radiomic features were used to construct the radiomics model after SVM regression and RFE feature selection, while 4 clinicopathological factors were selected to build the clinical model. For the merged model, 8 radiomic and 4 clinicopathological factors selected from the merged initial feature pool were included. Since each model was built by selecting features from the initial feature pool to achieve the best predictive performance, the features included in each model were different and uncorrelated. Table S2 in the Supplement presents detailed information on the features of each prediction model. The models were separately applied to predict the risk of ROM for each patient, and the *prob* was simultaneously calculated for further analysis.

### Performance of the RS-EPI DWI radiomics model and clinical model

**Fig. 2:**
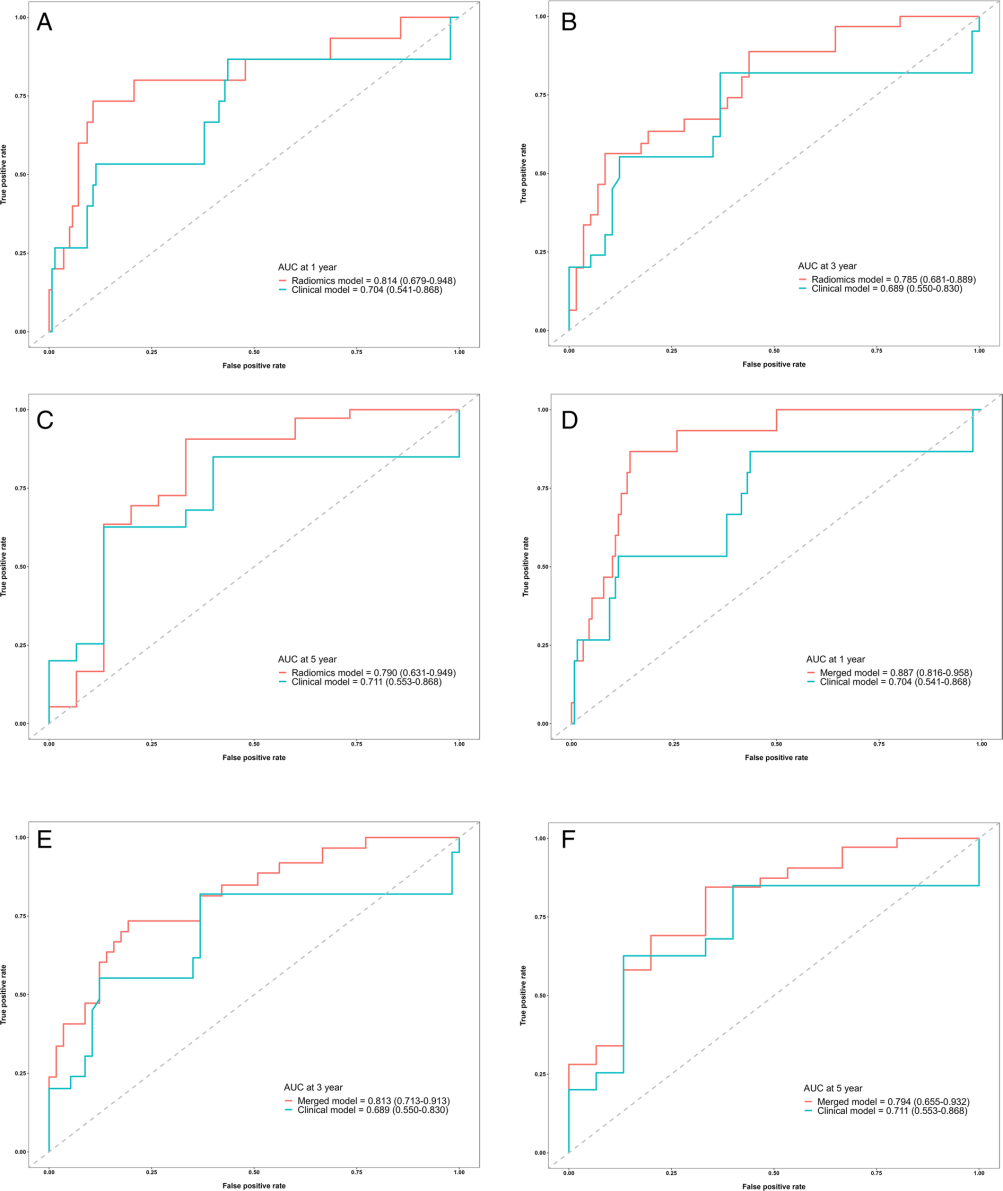

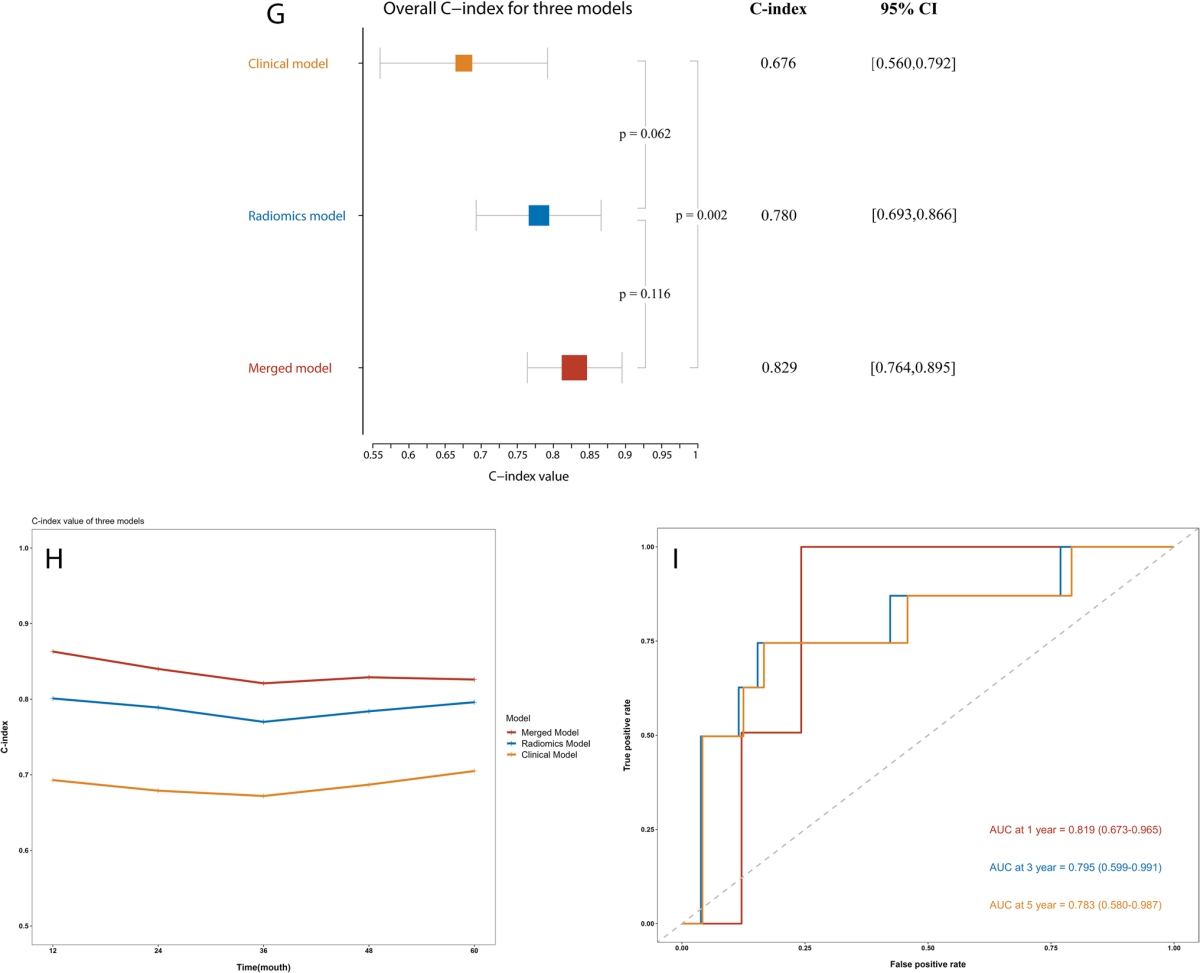
Assessment of the predictive performance of each constructed model for tumour recurrence or metastasis. **a**–**c** Comparisons of time-ROC curves between the radiomics model based on RS-EPI DWI and the clinical model **a** Time-ROC curves at 1 year. **b** Time-ROC curves at 3 years. **c** Time-ROC curves at 5 years. **d**–**f** Comparisons of time-ROC curves between the merged model and clinical model. **d** Time-ROC curves at 1 year. **e** Time-ROC curves at 3 years. **f** Time-ROC curves at 5 years. **g**–**h** Comparisons of C-index values for each model. **g** Forest plot showing the overall C-index values and 95% CIs of the three models: clinical model, 0.676 (95% CI: 0.560–0.792); radiomics model, 0.780 (95% CI: 0.693–0.866); and merged model, 0.829 (95% CI: 0.764–0.895). **h** The diagram shows the C-index value for each year of the 5-year period obtained using the three models: clinical model: 0.693, 0.679, 0.672, 0.687, and 0.705; radiomics model: 0.801, 0.789, 0.770, 0.784, and 0.796; and merged model: 0.863, 0.840, 0.821, 0.829, and 0.826. **i** External validation of the performance of the merged model with the time-ROC curves

We investigated whether the ML model based on the RS-EPI DWI radiomic features had better predictive performance for ROM than the model based on the clinicopathological factors by comparing the radiomics and clinical models. The radiomics model showed greater discrimination performance than the clinical model, with a higher AUC value for the time-ROC curve (1 year: 0.814 [95% CI: 0.679–0.948] vs. 0.704 [95% CI: 0.541–0.868]; 3 years: 0.785 [95% CI: 0.681–0.889] vs. 0.690 [95% CI: 0.550–0.830]; 5 years: 0.790 [95% CI: 0.631–0.949] vs. 0.711 [95% CI: 0.553–0.868]). The time-ROC curves of the two models are displayed in Fig. 2a-c. However, when comparing the overall AUC with the DeLong test, no statistically significant differences were observed between the models (AUC: 0.82 [95% CI: 0.74–0.90] vs. 0.71 [95% CI: 0.59–0.81], *p* = 0.118). Similar performance was also observed using the C-index. The radiomics model yielded higher C-index values (1 year: 0.801 vs. 0.693; 3 years: 0.770 vs. 0.672; 5 years: 0.796 vs. 0.705) at different time points. After using the R package “*survcomp*” to further compare the overall C-index, no statistically significant differences were identified (radiomics model vs. clinical model: 0.780 [95% CI: 0.693–0.866] vs. 0.676 [95% CI: 0.560–0.792], *p* = 0.062).

### Incremental value of the combination of radiomic features and clinicopathological factors

A comparison of the merged and clinical models was performed to investigate whether combining the extracted radiomic information with the clinicopathological information has incremental value compared to using the clinicopathological information alone. After combination, the merged model yielded AUC values of 0.887 (95% CI: 0.816–0.958), 0.813 (95% CI: 0.550–0.830), and 0.794 (95% CI: 0.655–0.932) and C-index values of 0.863, 0.821, and 0.826 at 1, 3, and 5 years, respectively. The aforementioned results for the merged model all indicated significantly better performance than the clinical model with a certain degree of improvement in the benchmark of the radiomics model. The time-ROC curves of the merged model and clinical model are presented in Fig. 2d-f. The overall values of AUC and C-index were 0.87 (95% CI: 0.80–0.93) and 0.829 (95% CI: 0.764–0.895), respectively. When compared to the values for the clinical model, both indicators showed statistically significant differences (*p* = 0.009; 0.002). The C-index of the three models is depicted in Fig. 2g-h, and DCA is depicted in Fig. S1 in the Supplement. The calibration curves (Fig. S2 in the Supplement) for the three models at 1, 3, and 5 years all showed great agreement between the estimates and the actual results. Table S3 in the Supplement lists the accuracy, sensitivity, and specificity of each model. The merged model outperformed the clinical model in terms of accuracy (85.4% vs. 75.2%) and specificity (88.1% vs. 74.6%) but slightly underperformed in terms of sensitivity (74.2% vs. 77.4%).

Moreover, the performance of the merged model was further validated for its consistency in cohort 2. It yielded AUC values of 0.819 (95% CI: 0.673–0.965), 0.795 (95% CI: 0.599–0.991), and 0.783 (95% CI: 0.580–0.987) at 1, 3, and 5 years, respectively, which are depicted in Fig. 2i.

### Risk stratification using the calculated risk probability

**Fig. 3:**
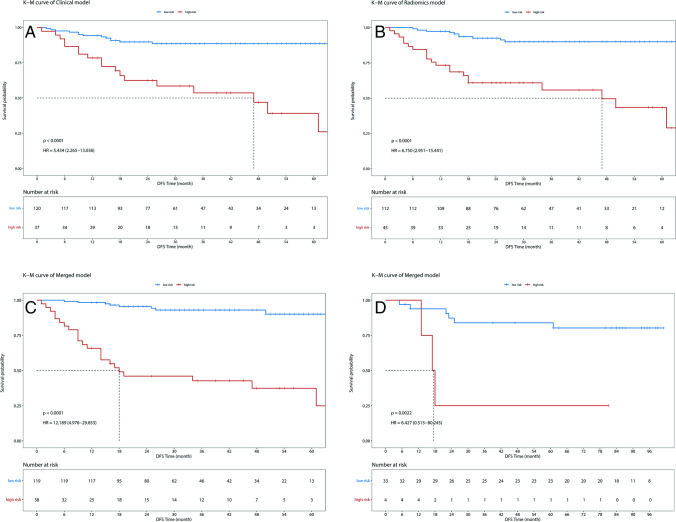
Comparisons of results from the K-M analysis for predicting DFS of patients with RC in our dataset. **a** K-M curve of the clinical model for cohort 1. **b** K-M curve of the radiomics model for cohort 1. **c** K-M curve of the merged model for cohort 1. **d** K-M curve of the merged model for cohort 2. *p* values were calculated using a two-sided log-rank test. HR: hazard ratio

The patients in the study were further stratified into two groups according to the *prob*. Those whose *prob* was greater than 0.5 were defined as having a high risk of ROM, while the remaining patients were defined as having a low risk. A comparison of the status of the outcome events between the high- and low-risk groups for each model was performed using K-M analysis, and the results of the survival analysis are shown in Fig. 3a-d. After using the log-rank test to evaluate significant differences between the two groups, the models all exhibited favourable distinguishability (*p* < 0.001). When comparing the HR of each model, the merged model (HR = 12.189, 95% CI: 4.976–29.853, *p* < 0.001) also showed a significant improvement over the radiomics model (HR = 6.750, 95% CI: 2.951–15.441, *p* < 0.001) and clinical model (HR = 5.434, 95% CI: 2.265–13.036, *p* < 0.001). For the results from cohort 2, the merged model maintained its good performance, with an HR value of 6.427 (95% CI: 0.515–80.245, *p* = 0.002).

**Fig. 4:**
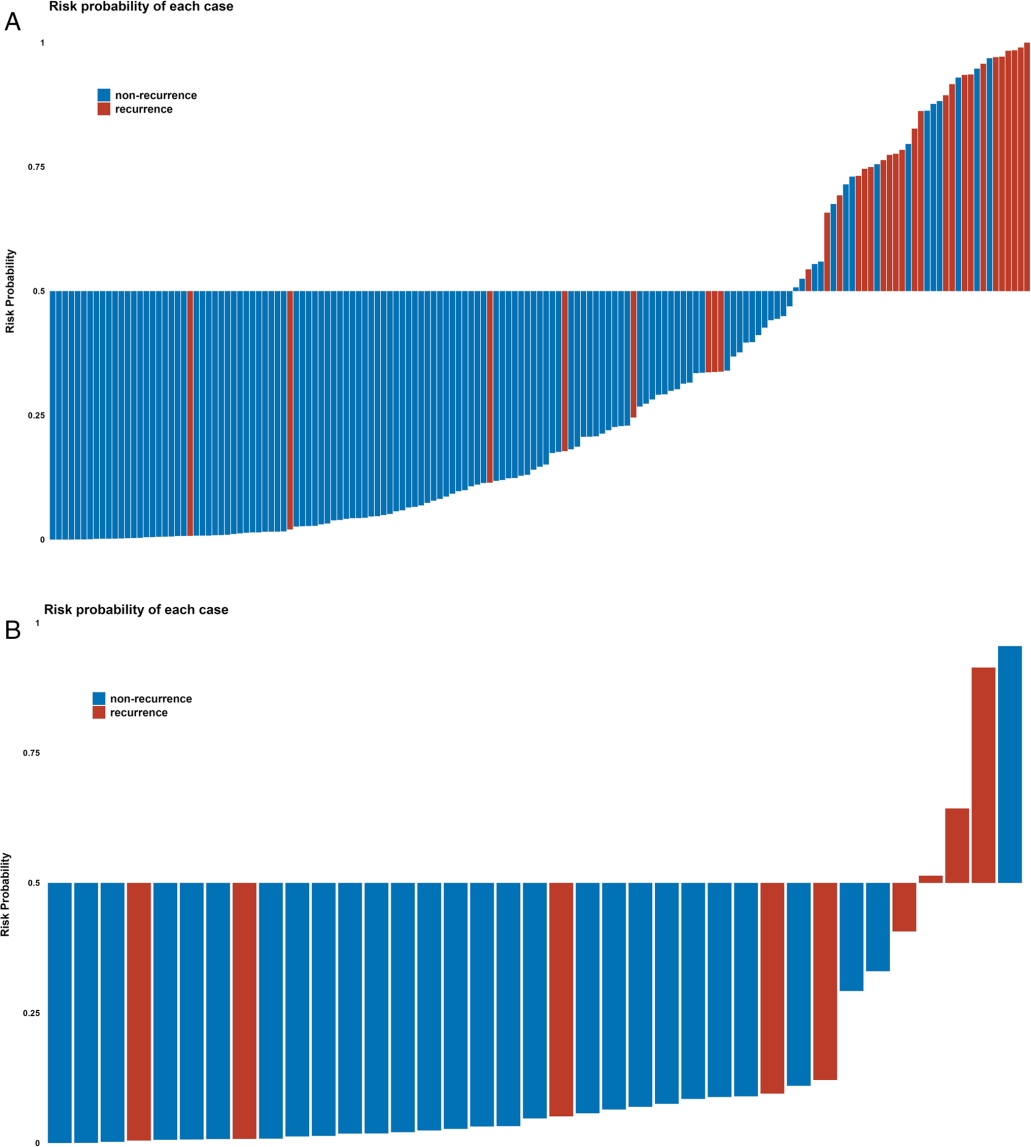
Risk probability calculated using the merged model for each patient with RC. Red bars represent the probability for patients who experienced ROM, while blue bars represent the probability for those who did not experience ROM. With a cut-off value of 0.5, bars above the x-axis represent patients who were stratified as having a high risk of ROM by the model, while bars below the x-axis represent patients who were stratified as having a low risk. Red bars above the x-axis or blue bars below the x-axis indicate cases where the results predicted by the model are consistent with the actual situation. **a** Patients from cohort 1 (135/158, 85.4%). **b** Patients from cohort 2 (30/37, 81.1%)

The risk probability distribution map (Fig. 4a-b) derived from the merged model of all patients in both cohorts indicated that the group result was highly consistent with the corresponding actual event status for most of the patients (135/158, 85.4%; 30/37, 81.1%).

## Discussion

Currently, the paradigm shift in cancer treatment from traditional reactive medicine to PPPM remains a hot topic. Using the PPPM approach, such as noninvasive imaging methods, to early and accurately predict the prognosis after treatment of each individual is a major facilitator of this shift, which is critical for the early adjustment of treatment protocols. This two-centre study was the first based on RS-EPI DWI to extract radiomic features from baseline data and integrate ML algorithms as a method to investigate its utility in predicting DFS of RC patients with direct surgery, culminating successfully in the development and validation of an optimal model. Our results revealed that the constructed radiomics model performed well in predicting the prognosis and stratifying patients according to the risk of ROM. Compared to the use of clinicopathological information alone, the integrated application of radiomic and clinicopathological information significantly improved the predictive capability of the model. Therefore, the proposed predictive model may support the implementation of this significant shift in RC treatment.

### Radiomics with the ML algorithm might provide essential additional information

Using the framework of PPPM, radiomics methods with ML algorithms might be a more effective approach than conventional morphology imaging. Due to tumour heterogeneity and differences in the microenvironment, variability in the outcome of treatment response and ROM for patients with RC consistently exists, which has been a great challenge for choosing the optimal protocol that avoids both under- and overtreatment. Researchers have increasingly focused on identifying image features as predictors for the long-term prognosis, which provides an essential foundation for the development of individualized medicine [20]. However, morphological manifestations indicating tumour progression are often extremely subtle and relatively lag in occurrence, indicating that they are difficult to detect on initial images through a traditional visual assessment [21]. Radiomics methodology enables the quantification of heterogeneity, which is inaccessible by the human eye, via high-throughput extraction of features hidden behind the radiology image and enables the construction of models for decision support.

One reason for the suboptimal adaptation of standard cancer treatment protocols may be the insufficient availability and utilization of information about the characteristics of the tumour itself. As an increasing number of features are developed, traditional statistical methods, such as logistic regression and multiple linear regression models, have become increasingly overwhelmed with such large and complex data. Among numerous other studies, ML algorithms have exhibited excellent performance in discovering potential connections from intricate data [12, 22, 23], facilitating the selection of the most suitable fraction from this vast amount of radiomic features beyond human understanding. Therefore, the accurate predictive ability of the model, a crucial aspect highlighted in the PPPM, might be guaranteed by the powerful data processing capacity of ML algorithms.

### The application of RS-EPI DWI radiomics showed great predictive accuracy 

The application of RS-EPI DWI to predict aggressive features, such as the status of EGFR and tumour differentiation, in RC has been reported in a limited number of previous studies [24–26]. Wen et al. further applied an ML method based on T2WI and RS-EPI DWI to predict the T stage of RC, which exhibited fairly high accuracy with AUCs of 0.893 and 0.810 in the training and testing cohorts, respectively. These findings revealed the great prognostic potential of this technique. However, to the best of our knowledge, this study was the first to apply radiomic features derived from RS-EPI DWI in predicting the ROM risk of patients with RC.

RS-EPI DWI provides remarkable advantages in image quality, geometric distortion, and discrimination of tissue variability, as we previously reported [27], generating a sharper visual boundary between the lesion and the surrounding normal tissue to improve the accuracy of manual lesion delineation and reducing the effect of image noise on high-dimensional features. Thus, the extracted features might be more representative of the actual tumour microenvironment. In a study predicting DFS, the radiomics model based on SS-EPI DWI was modestly evaluated by calculating the C-index in both the training (0.627, 95% CI%: 0.529–0.726) and test cohorts (0.658, 95% CI%: 0.536–0.779), while the performance of the model based on RS-EPI DWI in our study was substantially improved (C-index = 0.78 ± 0.04, 95% CI: 0.69–0.87), indicating that the technical superiority may indeed contribute to model optimization and was reflected in the forecasting results to some extent.

### The proposed models effectively stratify the ROM risk of RC patients and thus provide a reference for the application of PPPM

In previous studies a variety of clinicopathological features are intimately associated with the prognosis of RC patients [7, 8, 28, 29]. For example, Ceyhan et al. [28] suggested that PNI is useful as a prognostic factor for patients with RC. A nomogram model based on multiple clinicopathological factors, including pathological stage and adjuvant therapy, was proven to be effective in predicting ROM [29]. These findings were highly consistent with the key features of the clinical model selected by ML algorithms in our study, which supported the validity of our model construction approach.

Furthermore, some studies have proven the incremental value of integrating imaging and clinicopathological information for the performance of tumour assessment and prognostic prediction in patients with glioblastoma multiforme, advanced nasopharyngeal carcinoma, breast cancer, lung cancer, prostate cancer, and RC [30, 31]. The same was true for the results of our study. In our study, no significant differences were observed between the radiomics model and clinical model, as evaluated using the AUC (*p* = 0.118) or the C-index (*p* = 0.062). However, after combining the two features, a significant improvement was observed in the overall performance of the merged model compared to the clinical model (AUC = 0.87 [95% CI: 0.80–0.93] vs. 0.71 [95% CI: 0.59–0.81], *p* = 0.009; C-index = 0.829 [95% CI: 0.764–0.895] vs. 0.676 [95% CI: 0.560–0.792], *p* = 0.002). Although the predictive ability inevitably decreased slightly as the interval between the baseline and prediction time points increased, it still maintained a comparatively high level of predictability at any point of 1, 3, and 5 years (AUC = 0.887 [95% CI: 0.816–0.958] vs. 0.704 [95% CI: 0.541–0.868], 0.813 [95% CI: 0.713–0.913] vs. 0.690 [95% CI: 0.550–0.830], 0.794 [95% CI: 0.655–0.932] vs. 0.711 [95% CI: 0.553–0.868]; C-index = 0.863 vs. 0.693, 0.821 vs. 0.672, 0.826 vs. 0.705, respectively). Regarding the clinical utility of the model, the results of DCA also revealed that the merged model potentially provided a significant increase in benefits compared to the clinical model, which further reflected the improved accuracy of the merged model. After external validation, the combined model continued to achieve high accuracy (AUC: 1 year = 0.819 [95% CI: 0.673–0.965], 3 years = 0.795 [95% CI: 0.599–0.991], and 5 years = 0.783 [95% CI: 0.580–0.987]). However, limited by the small number of samples and endpoint events (9/37), some of the baseline characteristics differed between the two cohorts. The general applicability of the model may require further validation in a larger dataset of patients from more centres.

In addition, the results from the K-M curve indicated that the merged model provided a favourable stratification of the risk of ROM, with HR values of 12.189 (95% CI: 4.976–29.853, *p* < 0.001) and 6.427 (95% CI: 0.515–80.245, *p* = 0.002), which had significant implications for the arrangement of pre- and postoperative sequential treatment protocols. Adjustments in therapeutic regimens may require extreme caution, particularly for those patients whose results of risk stratification according to standard guidelines are inconsistent with our model. Therefore, the implementation of accurate ROM risk stratification and targeted pre-emptive interventions before poor prognostic outcomes occur for RC patients could be achieved using our proposed model, which represents the achievement of a momentous shift from delayed intervention to the PPPM treatment paradigm.

### Study strengths and limitations

We have developed certain innovations in the modelling approach to optimize the predictive power of the model. For the merged model, we adopted the same approach as for the radiomics model and clinical model, filtering the useful prognostic features from the pool that fused all clinicopathological factors and radiomic features directly, rather than by fusing the two models using a linear method such as a weighted average fusion strategy [32]. Our approach defined the clinicopathological and imaging information as the same dimension, thus reducing missing information in the data downscaling process and eliminating the effect on the performance due to the differences in the modelling methods. Differences in the selected features may imply the surrogate availability of certain clinicopathological factors and radiomic features for each other, consistent with our objectives in developing the radiomics profile to some extent.

Several limitations still exist in our study. First, since the RS-EPI DWI sequence is a new technique that has only been applied in the clinic for a short period, our sample size is relatively small, which partially contributed to the differences in baseline characteristics between the two cohorts. Although we have endeavoured to maximize the utilization of samples and prevent overfitting of the model using the LOOCV method, a larger sample is still required to further investigate the model for universal applicability. We are currently developing an advanced self-adaptive model that can automatically adjust model parameters as more criteria-compliant cases from different centres are continuously incorporated, thus constantly improving the versatility of the model. Second, the retrospective nature of patient enrolment inevitably leads to selection bias, which partially contributed to the differences in baseline characteristics between the two cohorts. Thus, prospective research will still be needed in the future. Third, regarding the factor of postoperative adjuvant therapy, we only focused on whether the patient had received it and did not explore the detailed protocol, which has been reported to be a valid DFS risk factor. We will further explore the aforementioned problem in our future studies. In addition to the protocol of manually contouring the lesions, which varied according to the clinical experience of the reviewers and image quality, the compatibility of image pathology remains a considerable obstacle to be overcome, although the image quality of RS-EPI DWI applied in our study was significantly improved compared to conventional DWI. In future research, we will further introduce premium methods such as deep learning algorithms to make improvements that might solve the aforementioned problems. Finally, the low interpretability of radiomics results continues to be the major obstacle to its progression.

## Conclusions and expert recommendations

In conclusion, we developed and validated a novel ML prediction model applying RS-EPI DWI-based radiomic features and clinicopathological factors from baseline data in our investigation, which supports the further development and application of PPPM in RC therapy. The continuing relatively high rate of ROM in patients with RC is closely related to the insufficient and delayed stratification of risk with current methods. Our model enables increased accurate prediction of the ROM risk of RC patients at an early stage, providing clinicians with essential additional information for treatment planning and follow-up management strategies to improve the overall prognosis of patients. The predictive and personalized nature of the model facilitates an influential paradigm shift in RC treatment from traditional reactive medicine to PPPM.

Given the findings of our study, we highly recommend that the methodology of radiomics with ML is applied in PPPM-relevant areas of cancer. Tumour heterogeneity remains a major obstacle for improving therapeutic efficacy and long-term prognosis. Quantitative radiomic features might reflect the microbiological, not only morphological, characteristics of the tumour for each individual, and ML enables the discovery of potential associations and the construction of valid biomarkers from these features. Since medical imaging has become one of the essential examinations in the process of diagnosis and treatment for various tumours, this information of tumours at multiple stages can be obtained easily and noninvasively, enabling radiomics methods to serve as a routine clinical assessment tool. Therefore, this method will definitely show great potential in future anticancer processes. By developing different key biomarkers, we can implement a more detailed classification of patients, which is critical for personalized management and targeted preventive interventions for cancers. In addition, we should actively explore improvements in predictive models by implementing new technologies, including both the fields of medical imaging and artificial intelligence. In the present study, the RS-EPI DWI-based model improved the predictive performance of the model for the ROM risk of patients with RC. With the development of more new technologies, such as various functional MRI sequences and deep learning algorithms, more information hidden in tumours that is currently unidentifiable will gradually be uncovered. A more comprehensive understanding of tumour biology will certainly facilitate the practical implementation of targeted and personalized medicine. Therefore, we believe that assessments and prediction models based on the methodology of radiomics with ML are effective strategies to provide personalized management of tumours in the context of PPPM.

## Supplementary Information

Below is the link to the electronic supplementary material.

Supplementary file1 (DOCX 683 KB)

## Data Availability

Due to the privacy of patients, the clinical data related to patients and MRI images cannot be available for public access but can be obtained from the corresponding author on reasonable request.

## Abbreviations

*RC*: Rectal cancer
*ROM*: Local recurrence or distant metastasis
*ML*: Machine learning
*MRI*: Magnetic resonance imaging
*PPPM*: Predictive, preventive, and personalized medicine
*DWI*: Diffusion-weighted imaging
*ADC*: Apparent diffusion coefficients
*PCR*: Pathological complete response
*OS*: Overall survival
*DFS*: Disease-free survival
*SS-EPI*: Single shot echo-planar imaging
*RS-EPI*: Readout-segmented echo-planar imaging
*CEA*: Carcinoembryonic antigen
*CA-199*: Carbohydrate antigen 199
*pT stage*: Pathological T stage
*pN stage*: Pathological N stage
*PNI*: Perineural invasion
*ROIs*: Regions of interest
*SVM*: Support vector machine
*RFE*: Recursive feature elimination
*SMOTE*: Synthetic minority oversampling technique
*LOOCV*: Leave-one-out cross-validation
*IQR*: Interquartile range
*Time-ROC*: Time-dependent receiver operating characteristic
*AUC*: Area under the ROC curve
*C-index*: Harrell concordance index
*DCA*: Decision curve analysis
*K-M*: Kaplan‒Meier
*CI*: Confidence interval
