# Supplementary material for: Radiomics based on readout-segmented echo-planar imaging (RS-EPI) diffusion-weighted imaging (DWI) for prognostic risk stratification of patients with rectal cancer: a two-centre, machine learning study using the framework of predictive, preventive, and personalized medicine
Source: EPMA J. 2022 Nov 12;13(4):633–47. doi: 10.1007/s13167-022-00303-3 (PMC9727035; doi:10.1007/s13167-022-00303-3)
Supplement: Supplementary file 1 — Supplementary file1 (DOCX 683 KB) [file 13167_2022_303_MOESM1_ESM.docx]

**Supplementary Figure：**


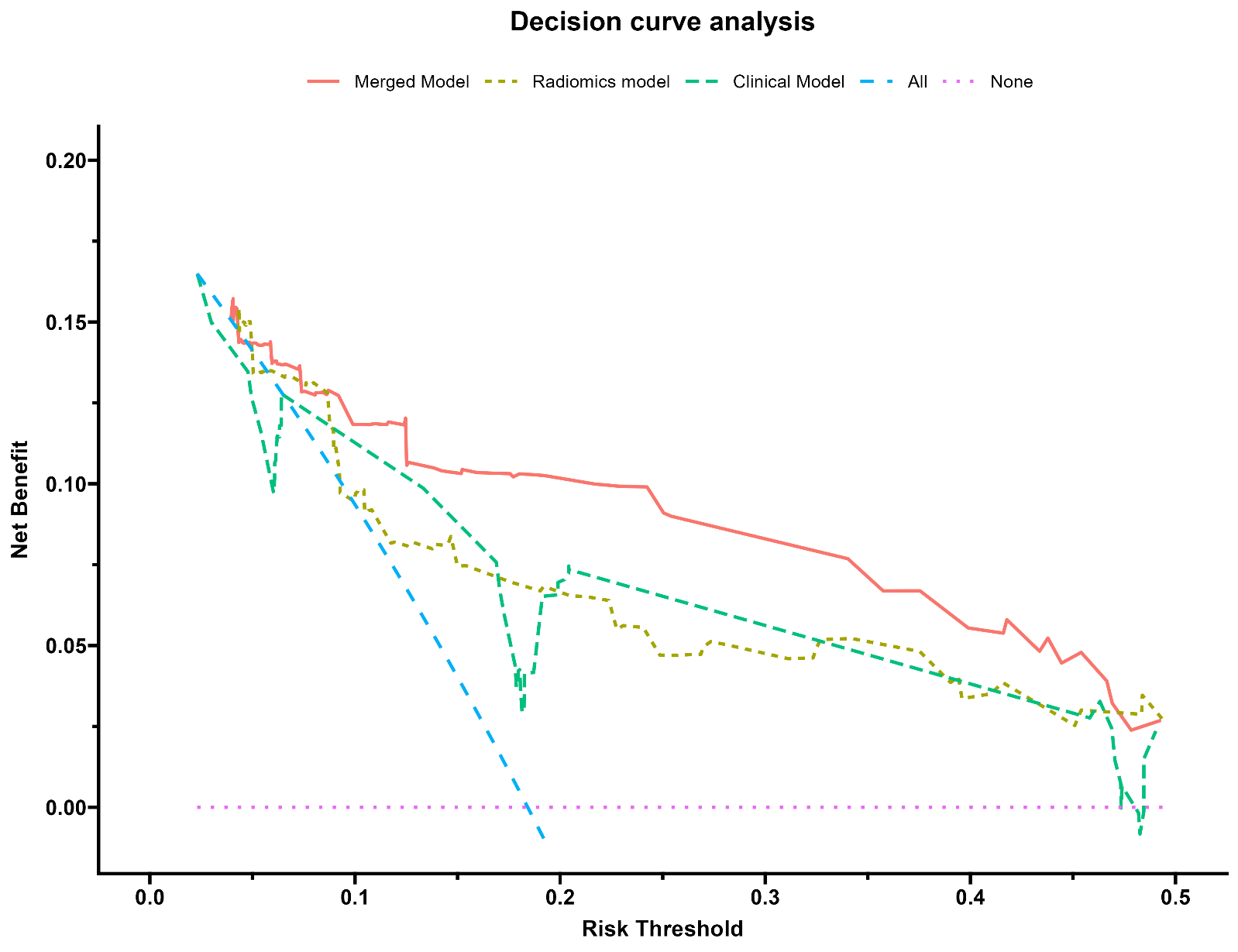


**Fig. S1.** Decision curve analysis at median time of DFS for the merged model, radiomics model, and the clinical model. The y-axis weighs the net benefit.


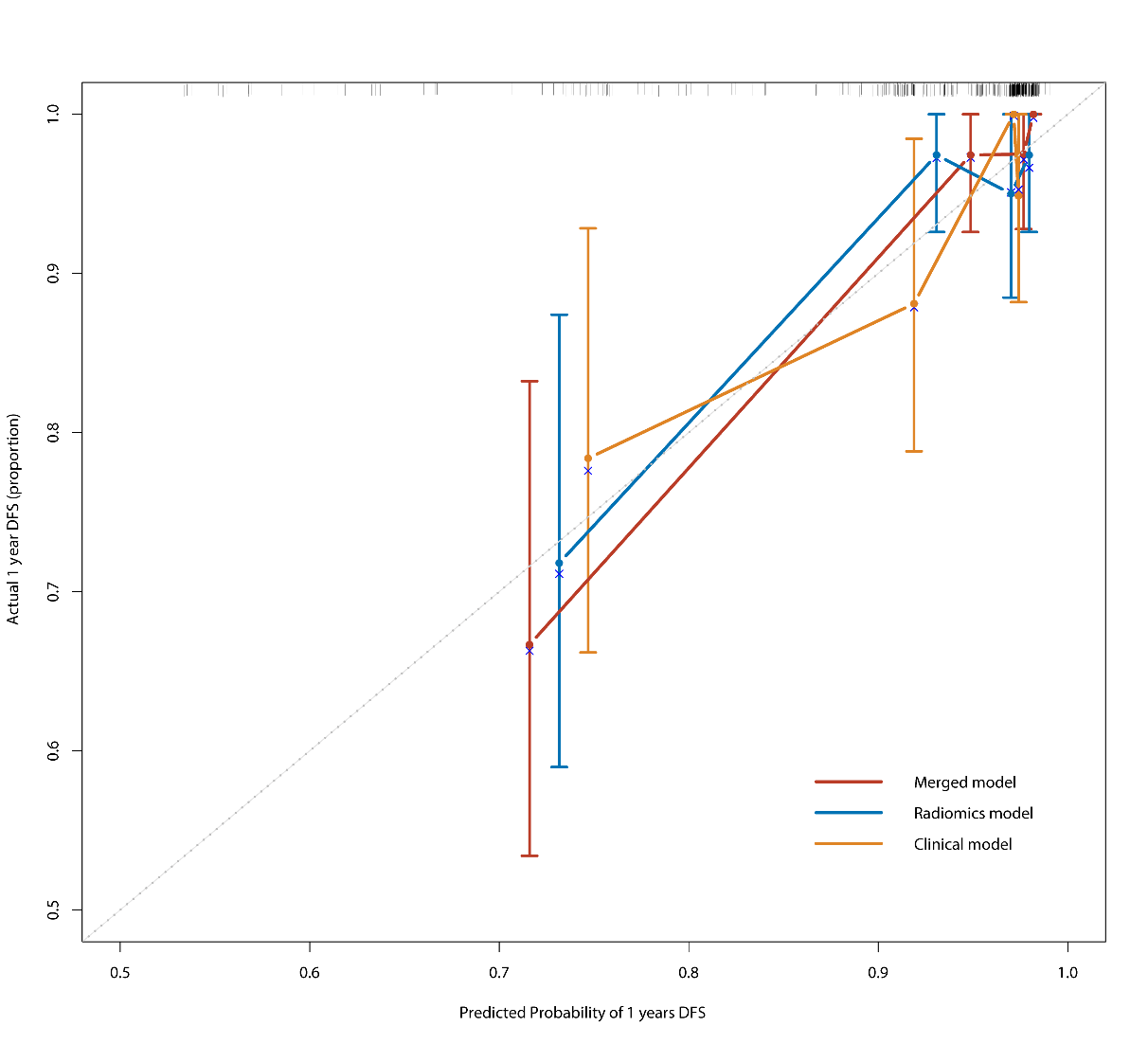

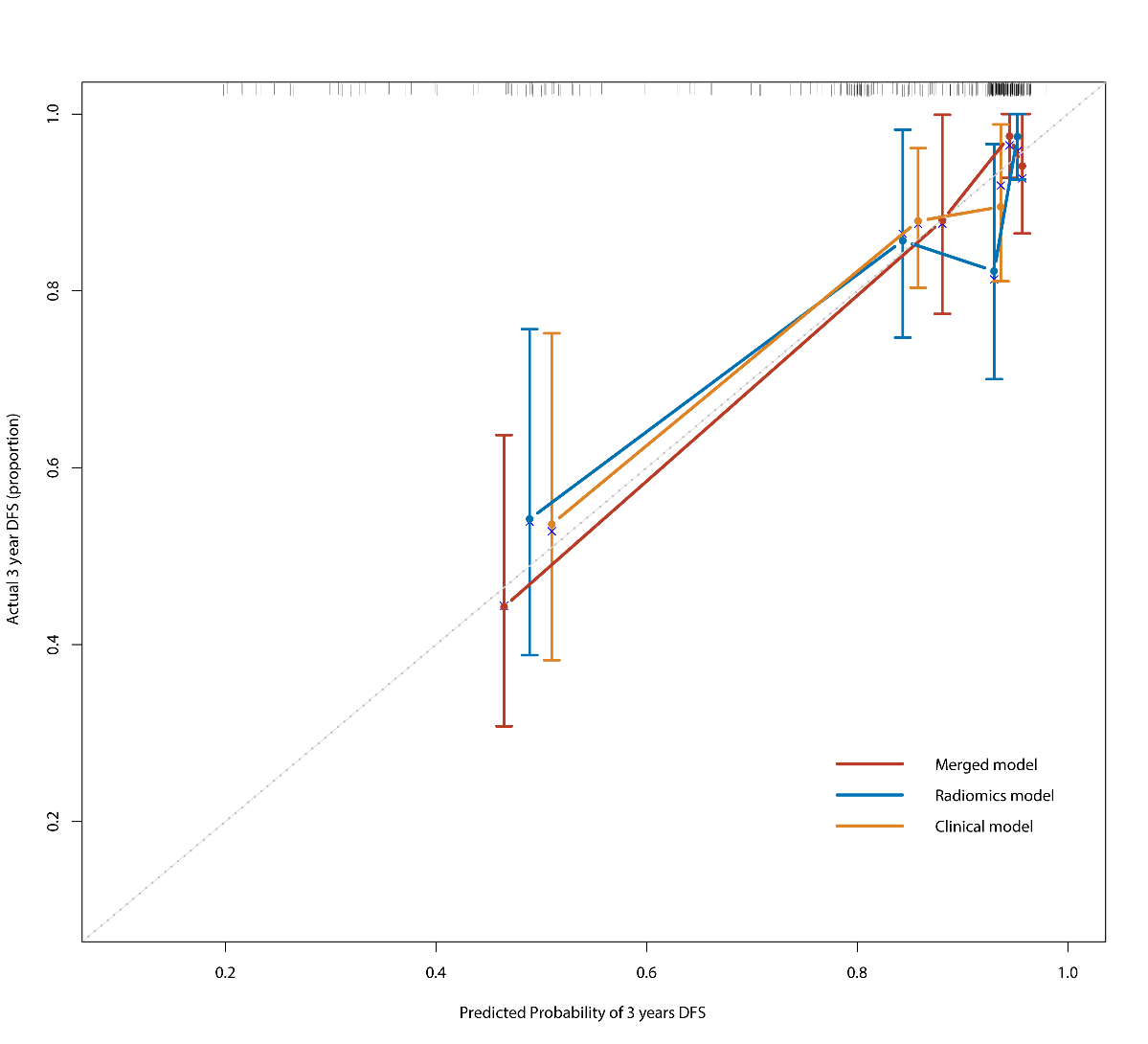

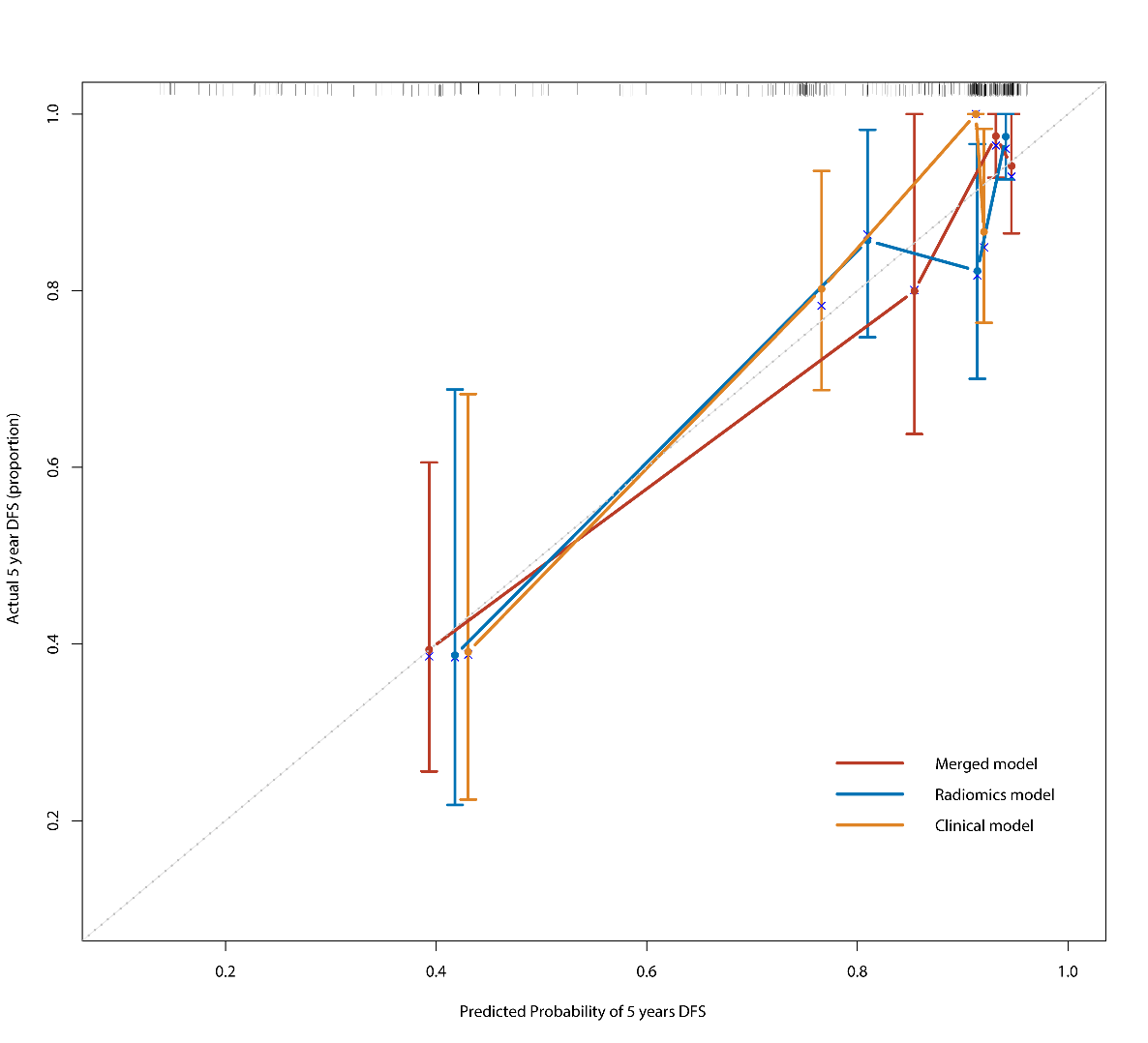


**Fig. S2.** Calibration curves of each model for evaluating DFS.

A. calibration curves of each model at 1 year. B. calibration curves of each model at 3 years. C. calibration curves of each model at 5 years. The error bars were defined as s.e.m., which represent the 95% CI. DFS disease-free survival.

**Supplementary Tables:**

**Table S1.** MRI scanner protocols and parameters for the two centers

|  | **FDSHCC** | | **SMMUCH** | |
| --- | --- | --- | --- | --- |
| Sequence | T2WI | RESOLVE | T2WI | RESOLVE |
| Scanning device (series, company) | MAGNETOM Skyra, Siemens | | MAGNETOM Skyra, Siemens | |
| Magnetic field Strength (T) | 3.0 | | 3.0 | |
| Sequence technique | TSE | RS-EPI | TSE | RS-EPI |
| TR/TE (msec) | 1500/100 | 4900/60 | 4000/108 | 5200/86 |
| Flip angle (°) | 135 | 180 | 160 | 180 |
| FOV (mm^2^) | 230*230 | 180*200 | 180*180 | 280*280 |
| Matrix | 320*291 | 140*126 | 320*320 | 170*120 |
| Nsa | 2 | 2 | 2 | 1 |
| Slice thickness (mm) | 1.5 | 5.5 | 3 | 5 |
| Slice number | 64 | 20 | 28 | 25 |
| Slice gap (mm) | 0 | 5.5 | 0 | 0 |
| Number of averages | 2 | 2 | 2 | 1 |
| Bandwidth (Hz/pixel) | 600 | 940 | 110 | 817 |
| B-values (s/mm^2^) |  | 0, 800 |  | 0， 1000 |

Abbreviations: a. FDSHCC: Fudan University Shanghai Cancer Center; b. SMMUCH: Chang Hai Hospital of Second Military Medical University. c. TSE: turbo spin-echo; d. RS-EPI: readout-segmented echo-planar imaging

**Table S2.** Selected features for model construction

|  | **Selected features** |
| --- | --- |
| Clinical model | ‘pT stage’  ‘pN stage’  ‘PNI’  ‘Post-operational adjuvant therapy’ |
| Radiomics model | 'Original_firstorder_InterquartileRange'  'Original_firstorder_Maximum'  'Original_firstorder_Median'  'Original_firstorder_Range'  'Original_glcm_DifferenceVariance'  'Original_glcm_InverseVariance'  'Log-sigma-3-0-mm-3D_glcm_Correlation'  'Log-sigma-3-0-mm-3D_glcm_Imc1'  'Log-sigma-3-0-mm-3D_glrlm_ShortRunEmphasis'  'Log-sigma-5-0-mm-3D_firstorder_Skewness'  'Log-sigma-5-0-mm-3D_glcm_ClusterProminence'  'Wavelet-LLH_firstorder_Skewness'  'Wavelet-LHH_firstorder_Skewness'  'Wavelet-HLH_glcm_DifferenceVariance'  'Wavelet-HHL_gldm_DependenceVariance' |
| Merged model | 'Original_firstorder_Maximum'  'Original_glcm_DifferenceVariance'  'Log-sigma-3-0-mm-3D_glcm_InverseVariance'  'Log-sigma-5-0-mm-3D_firstorder_Skewness'  'Log-sigma-5-0-mm-3D_glcm_ClusterProminence'  'Wavelet-HLH_firstorder_InterquartileRange'  'Wavelet-HLH_glcm_ClusterTendency'  'Wavelet-HLH_glcm_DifferenceEntropy'  'CEA'  'CA 19-9'  'Tumor differentiation'  'Intravenous tumor emboli' |

Abbreviations: a. pT stage: pathological T stage, b. pN stage: pathological N stage, c. PNI: perineural invasion, d. nCRT: neoadjuvant radiotherapy, e. CEA: carcinoembryonic antigen, f. CA19-9: carbohydrate antigen 19-9.

**Table S3.** Comparison of Accuracy, Sensitivity and Specifity of each model

| **Model** | **Overall AUC (95%CI)** | **Accuracy (%)** | **Sensitivity (%)** | **Specifity (%)** |
| --- | --- | --- | --- | --- |
| Clinical model | 0.71 (0.59-0.81) | 75.2 | 77.4 | 74.6 |
| Radiomics model | 0.82 (0.74-0.90) | 78.3 | 67.7 | 81.0 |
| Merged model | 0.87 (0.80-0.93) | 85.4 | 74.2 | 88.1 |
